# Fibroblastome à cellules géantes inhabituel: à propos d’un cas

**DOI:** 10.11604/pamj.2017.28.263.12766

**Published:** 2017-11-24

**Authors:** Mohammed Boukhechba, Najat Lamalmi, Abderrahmane Malihy, Lamia Rouas, Zaitouna Al Hamany

**Affiliations:** 1 Service d’Anatomie Pathologique, Hôpital d’Enfants, 18, Avenue Attine, Secteur 7, Hay Riad, Rabat, Maroc

**Keywords:** Fibroblastome, cellules géantes, enfant, Fibroblastoma, giant cells, infant

## Abstract

Le fibroblastome à cellules géantes (FCG) est une tumeur rare de l'enfant de sexe masculin et de localisation superficielle. Nous rapportons un nouveau cas observé chez un enfant de 3 mois qui avait consulté pour une masse du bras droit, une IRM du bras avait montré une masse de siège sous cutané, cette masse a été réséquée de façon complète. Les études histologique, immuno-histochimique et génétique avaient conclu au diagnostic d'un FCG. Cette tumeur pose des difficultés diagnostiques pour le pathologiste avec les tumeurs mésenchymateuses malignes de pronostic différent.

## Abstract (en)

Giant cell fibroblastoma (GCF) is a rare superficial tumor occurring in male infants. We report the case of a 3-month old infant examined for a mass in his right arm. Arm MRI objectified a subcutaneous mass. The patient underwent total excision of the mass. Histological, immuno-histochemical and genetic examinations showed giant cell fibroblastoma. This tumor poses diagnostic challenges to the pathologist because it may be confused with malignant mesenchymal tumors with different prognosis.

## Introduction

Le fibroblastome à cellules géantes est une tumeur mésenchymateuse rare de malignité intermédiaire décrite en 1982 par Shmookler et Enzinger dans une série de vingt cas [1]. C'est une tumeur qui survient chez l'enfant au niveau de la cuisse, du dos, des extrémités, de la tête et du cou, rarement dans la région génitale [2]. Les métastases sont absentes, mais les récidives locales sont fréquentes et se voient dans la moitié des cas [1-7]. Nous rapportons un nouveau cas observé chez un enfant qui a présentait des difficultés diagnostiques vue son aspect histologique particulier riche en lymphoplasmocytes et polynucléaires éosinophiles illustrant ainsi le rôle du pathologiste dans le diagnostic positif de cette entité en se basant sur l'histologie couplée à l'immunohistochimie, parfois une étude génétique s'avère nécessaire pour éliminer les diagnostics différentiels.

## Patient et observation

**Figure 1:**
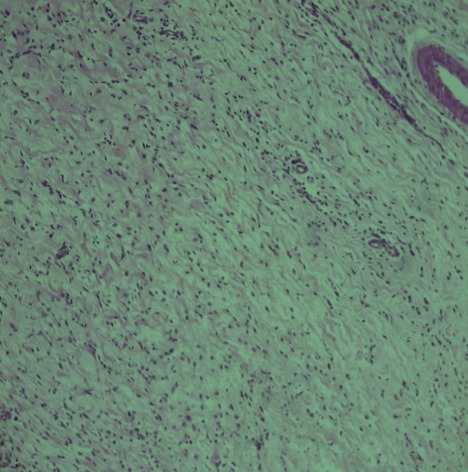
Coloration HE (grossissement × 10). Territoires myxoïdes et pauci cellulaires avec un infiltrat inflammatoire lymphocytaire à disposition caractéristique périvasculaire en bulbe d’oignon

**Figure 2:**
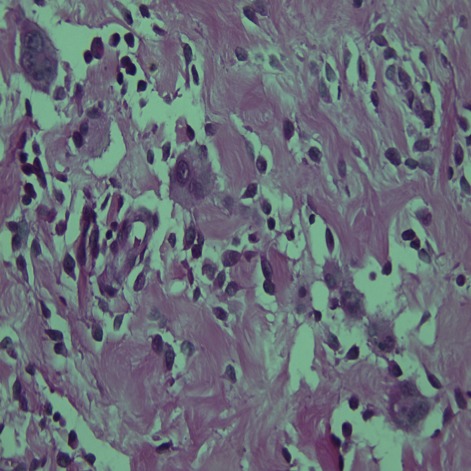
Coloration HE (grossissement × 20). Fond fibreux comportant des faisceaux de collagène épais et hyalins, entre ces faisceaux s’interposent des cellules fusiformes, et des cellules géantes pléomorphes particulièrement autour des fentes pseudovasculaires, on note également l’infiltrat inflammatoire polymorphe lymphoplasmocytaire et à polynucléaires éosinophiles

**Figure 3:**
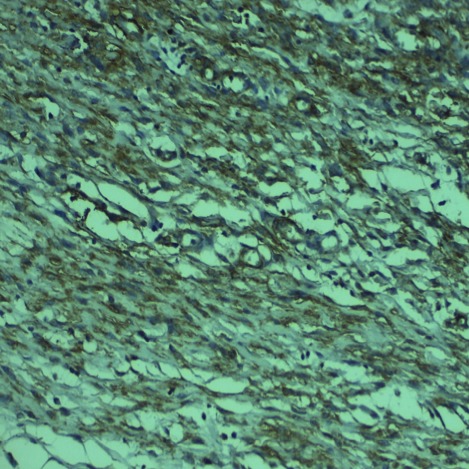
Immunohistochimie (grossissement x 40). Les cellules tumorales expriment le CD34

Enfant de 3 mois qui a consulté pour une masse du bras droit augmentant progressivement de volume avec conservation de l'état général, l'examen clinique a trouvé une masse de consistance ferme et indolore mesurant 6cm de grand axe. Une IRM a objectivé une tumeur sous cutané adhérant intimement au muscle biceps brachial. Cette masse a été réséquée et l'examen au microscope optique a montré une prolifération tumorale de densité cellulaire variable avec l'alternance de zones myxoides (Figure 1) et d'autres fibreuse (Figure 2), les cellules tumorales sont fusiformes assez monomorphes aux cytoplasmes peu abondant, aux noyaux ronds ne montrant ni atypies cytonucléaires ni mitoses, il s'y associé la présence de cellules géantes d'allure bizarre bordant des fentes pseudovasculaires (Figure 2), un fait particulier était la présence d'un infiltrat inflammatoire polymorphe riche en lymphoplasmocytes et leucocytes éosinophiles préférentiellement autour des vaisseaux (Figure 2) portant confusion avec une tumeur myofibroblastique inflammatoire (TMI). Il n'a pas été vu de nécrose, ni d'invasion vasculaire. L'étude immuno-histochimique a montré un marquage franchement positif par l'anticorps anti-CD34 (Figure 3); les cellules tumorales n'exprimaient pas le CD99, la PS100, l'actine muscle lisse, la desmine et la myogénine, l'ALK été aussi négatif permettant ainsi d'éliminer une TMI. Le diagnostic était confirmé par la mise en évidence du gène de fusion COL1A1-PDGFB en utilisant une technique d'hybridation fluorescente in situ.

## Discussion

Le fibroblastome à cellules géantes est une tumeur rare du tissu mou qui appartient aux lésions d'origine fibrohistiocytaire à malignité intermédiaire [1, 2]. C'est une tumeur intéressant surtout l'enfant comme dans l'observation rapportée avec un âge médian de 6 ans [1-4], La survenue après 40 ans est rare [3]. La prévalence est deux fois plus élevée dans le sexe masculin [1-4]. La tumeur survient préférentiellement dans la cuisse, le dos et la paroi thoracique [1-3], Des localisations plus exceptionnelles ont été signalées: creux axillaire, tête et cou et les extrémités comme dans cette observation [2, 3]. Cette tumeur est habituellement superficielle siégeant au niveau du derme et l'hypoderme [2-4]. La présentation clinique habituelle est l'apparition d'une masse sous-cutanée, indolore, d'évolution lente [1, 3-7]. Macroscopiquement, la tumeur apparaît mal limitée, d'aspect parfois mucoïde, de couleur blanc jaunâtre, La taille tumorale varie de 0,7 à 8cm [2, 4]. Microscopiquement, la tumeur est faite de zones fibreuses denses coexistant souvent avec des zones pauci cellulaires ou myxoïdes, un trait distinctif de cette tumeur est la présence d'espaces pseudovasculaires bordés par une rangée discontinue de cellules multinucléées pléomorphes en florettes. Dans la plupart des cas cette tumeur englobe les annexes cutanées sans les détruire. Il n'y a ni nécrose, ni invasion vasculaire, l'activité mitotique est rare. Une image qui paraît très particulière à cette tumeur et individualisée récemment est la présence quasi constante de lymphocytes autour des vaisseaux, prenant un aspect en bulbe d'oignon [4], dans notre cas il s'agit plutôt d'un infiltrat polymorphe riche en lymphocytes mais aussi en plasmocytes et polynucléaires éosinophiles. Sur le plan immunohistochimique, les cellules tumorales expriment le CD34, tout en étant négatif pour la protéine S-100, l'AML, l'ALK, la desmine, l'HMB-4^5^ et la cytokératine [2-4].

Sur le plan évolutif, La tumeur est caractérisée par un comportement localement agressif, des métastases n'ont pas été observées jusqu'à présent. La récidive après l'exérèse se voit dans presque la moitié des cas, celle ci peut prendre l'aspect d'un FCG ou d'un dermatofibrosarcome de Darier et Ferrand (DFS) même à l'âge adulte [2, 4]. L'histogenèse du FCG des parties molles n'est pas complétement élucidée, il partage certaines caractéristiques morphologiques, immunohistochimiques avec le dermatofibrosarcome de Darier et Ferrand (DFS) [2, 4]. Le FCG est considéré par certains auteurs comme la forme pédiatrique du dermatofibrosarcome de Darier et Ferrand [1]. Sur le plan génétique, le FCG et le DFS comportent la même la signature cytogénétique qui est une translocation t (17;22) (q22;q13) qui donne un gène hybride de la fusion des gène COL1A1-PDGFB. Ces caractéristiques génomiques et moléculaires permet un diagnostic de certitude en cas de doute [2-7]. Le diagnostic différentiel peut se poser avec le fibrosarcome infantile (FSI) et la tumeur myofibroblastique inflammatoire (TMI) comme dans cette observation. Une inflammation périvasculaire disposée en « pelures d'oignons », des espaces pseudovasculaires et des cellules géantes autour sont des arguments en faveur du FCG [3]. L'absence de la translocation caractéristique t(12;15) (p13;q25) produisant le gène de fusion ETV^6^-NTRK3 permet d'éliminer un FSI. La TMI présente un marquage positif à l'ALK. Le liposarcome peut également poser des problèmes diagnostiques, surtout lorsque des cellules géantes sont présentes dans les zones d'infiltration graisseuses mimant alors les lipoblastes. Pourtant le liposarcome est souvent plus profond et survient chez des sujets plus âgés [4]. Le traitement du fibroblastome à cellules géantes est chirurgical, consistant en une exérèse large, une surveillance postopératoire est obligatoire vu le caractère récidivant de cette tumeur [3, 4].

## Conclusion

Le fibroblastome est une tumeur rare de risque de malignité intermédiaire qui pose au pathologiste des problèmes de diagnostic différentiel. Les données de la cytogénétique sont d'un grand apport pour poser le diagnostic pour les cas difficiles. Par ailleurs il nécessite une longue surveillance vue son caractère récidivant.

## Conflits d’intérêts

Les auteurs ne déclarent aucun conflit d'intérêts.
